# Craniofacial morphology does not support a pre-contact Carib “invasion” of the northern Caribbean

**DOI:** 10.1038/s41598-021-95558-7

**Published:** 2021-08-20

**Authors:** Christina M. Giovas, Scott M. Fitzpatrick, Thomas P. Leppard, Mark Hubbe, William J. Pestle, Peter E. Siegel, L. Antonio Curet, José R. Oliver, Arie Boomert, Richard T. Callaghan

**Affiliations:** 1 Department of Archaeology, Simon Fraser University, Education Building 9635, 8888 University Dr., Burnaby, BC V5A 1S6 Canada; 2 Department of Anthropology, 1218 University of Oregon, 308 Condon Hall, Eugene, OR 97403 USA; 3 Museum of Natural and Cultural History, University of Oregon, Eugene, OR 97403 USA; 4 Department of Anthropology, Florida State University, Johnson Building, 2035 E. Paul Dirac Drive, Tallahassee, FL 32310 USA; 5 Aarhus Institute of Advanced Studies, Aarhus University, 8000 Aarhus, Denmark; 6 Department of Anthropology, The Ohio State University, Columbus, OH 43210 USA; 7 Instituto de Arqueología Y Antropología, Universidad Católica del Norte, San Pedro de Atacama, Chile; 8 Department of Anthropology, University of Miami, Coral Gables, FL 33124 USA; 9 Department of Anthropology, Montclair State University, Montclair, NJ 07043 USA; 10 Center for Heritage and Archaeological Studies, Montclair State University, Montclair, NJ 07043 USA; 11 National Museum of the American Indian, Smithsonian Institution, Washington, DC 20560 USA; 12 Institute of Archaeology, University College London, London, WC1H 0PY UK; 13 Faculty of Archaeology, Leiden University, Einsteinweg 2, 2333CC Leiden, The Netherlands; 14 Department of Anthropology and Archaeology, University of Calgary, Calgary, AB T2N 1N4 Canada

**Keywords:** Biological techniques, Environmental social sciences

## Introduction

**arising from**: A. H. Ross et al.;* Scientific Reports*10.1038/s41598-019-56929-3 (2020).

Ross et al.^1^ report results from the analysis of 103 pre-Columbian crania from 10 localities within the insular and circum-Caribbean. Based on geometric morphometric (GMM) and hierarchical cluster analyses, the authors conclude that individuals from Hispaniola (n = 15), Jamaica (n = 7), and the Bahamas (n = 8) form a distinct cluster (HJB) representing the migration of Caribs (peoples belonging to the Cariban language family) into the region, ca. AD 800. Ross et al.^1^ link the HJB cluster to a western Venezuelan homeland based on ostensible similarities between Carib pottery found in that region and the Meillacoid ceramics archaeologically associated with HJB populations. A second cluster comprising Cuba (n = 21) and the Yucatán (n = 12) and a third consisting of Puerto Rico (n = 10), Venezuela (n = 4), and Colombia (n = 5) are linked to continental emigration events at ca. 5000 BC and ca. 800–200 BC, respectively. Specimens from Florida (n = 15) and Panama (n = 6) form a fourth cluster. To support the ethnic distinctiveness of the Carib migrants/HJB cluster, the authors rely on Columbus’ account of cannibal marauders, identified as “Caribs”, attacking peaceful Arawakan-speaking communities of the Bahamas. Ross et al.’s^1^ study contains important shortcomings that bear on the reliability of their conclusions. We discuss the most significant issues here, and based on these we conclude there is no evidence to substantiate a Carib migration from Venezuela to Hispaniola 700 years before Columbus’ arrival.

The critical weaknesses of the paper lie in the number, chronology, and archaeological contexts of the crania examined. Substantiation of Ross et al.’s^1^ proposed migrations requires cranial specimens from a given island cluster to be appropriately associated in time and space with the continental specimens argued to represent source populations. It also requires the latter to be securely assigned to the proposed source culture based on archaeological context. The authors do not meet any of these evidentiary requirements. The four individuals from Venezuela, for instance, are of unknown age, location, and cultural affiliation and are unlikely to represent the full biological diversity and (presumably) associated ethnic and linguistic diversity of this region. The importance of considering archaeological context is critically exemplified by the 12 Chichén Itzá samples. Although not reported by Ross et al.^1^, at least 11 of these derive from the Sacred Cenote, a Maya human sacrificial context (ca. AD 800–1200) recently shown by Sr^87^/Sr^86^ and δ^18^O analyses to contain high proportions of non-local individuals, some possibly originating from as far away as Central America and Mexico’s Central Highlands^2^. Two samples from Ross et al. (SI^1^ accession numbers 58219, 58220; recorded as 07–7–20/58219.0 and 07–7–20/58220.0, Harvard Peabody Museum online catalogue) analyzed in that study are consistent with local origins, but the remaining eight, including two reported subadults (07–7–20/58203.0, 07–7–20/58225.0), are unaccounted for and may not represent local individuals and associated craniofacial traits.

Overall, 88 specimens (85%) lack site-specific provenience information and most lack direct radiocarbon dates (Table 2 and SI in Ross et al.^1^). They can be assigned only to 500–700 year ranges post-dating AD 600, or to the pre-contact period more generally. The uncertain spatial and temporal context of some specimens and absence of full details for others makes the sample inadequate for testing the proposed Carib-HJB migration.

Besides sample limitations, the paper employs statistical analysis that cannot reliably represent biological relationships between the series. Ross et al.^1^ identify three migrations based on four observed clusters in the morphological series produced by hierarchical cluster analysis. Traditionally used to reconstruct phylogenetic histories^3^, cluster analysis is limited as a method for inferring population histories, particularly for groups or populations that are biologically similar. Different clustering algorithms may result in disparate results^4^ and, importantly, clusters may create artificial associations between distant series because series are clustered one pair at a time^5^. To illustrate these issues and their impact on Ross et al.’s^1^ conclusions, we present the results of a Kruskal’s Non-Metric Multidimensional Scaling Analysis (MDS)^6^, computed with R^7^ using package MASS^6^, based on the Mahalanobis Distances reported in Ross et al.^1^ Table 3. This MDS analysis does not presume the existence of clusters among the data and calculates iteratively the closest possible bi-dimensional (or three-dimensional) visual representation of the distance matrix. The MDS reported here represents the data in two dimensions, using the centroid of the points as its origin^6^. The difference between observed distances and point positions in the final MDS output is measured through the stress in the analysis, measured in percentage, where a stress of 0 means the positions of the plotted points represent the distances in the matrix without any distortion. While MDS may result in distorted relationships between series since it attempts to depict multivariate space in two dimensions, in this case it represents more accurately the morphological distances between series (Ross et al.^1^ Table 3) and, as we will demonstrate, should be favored over hierarchical clustering.

**Figure 1:**
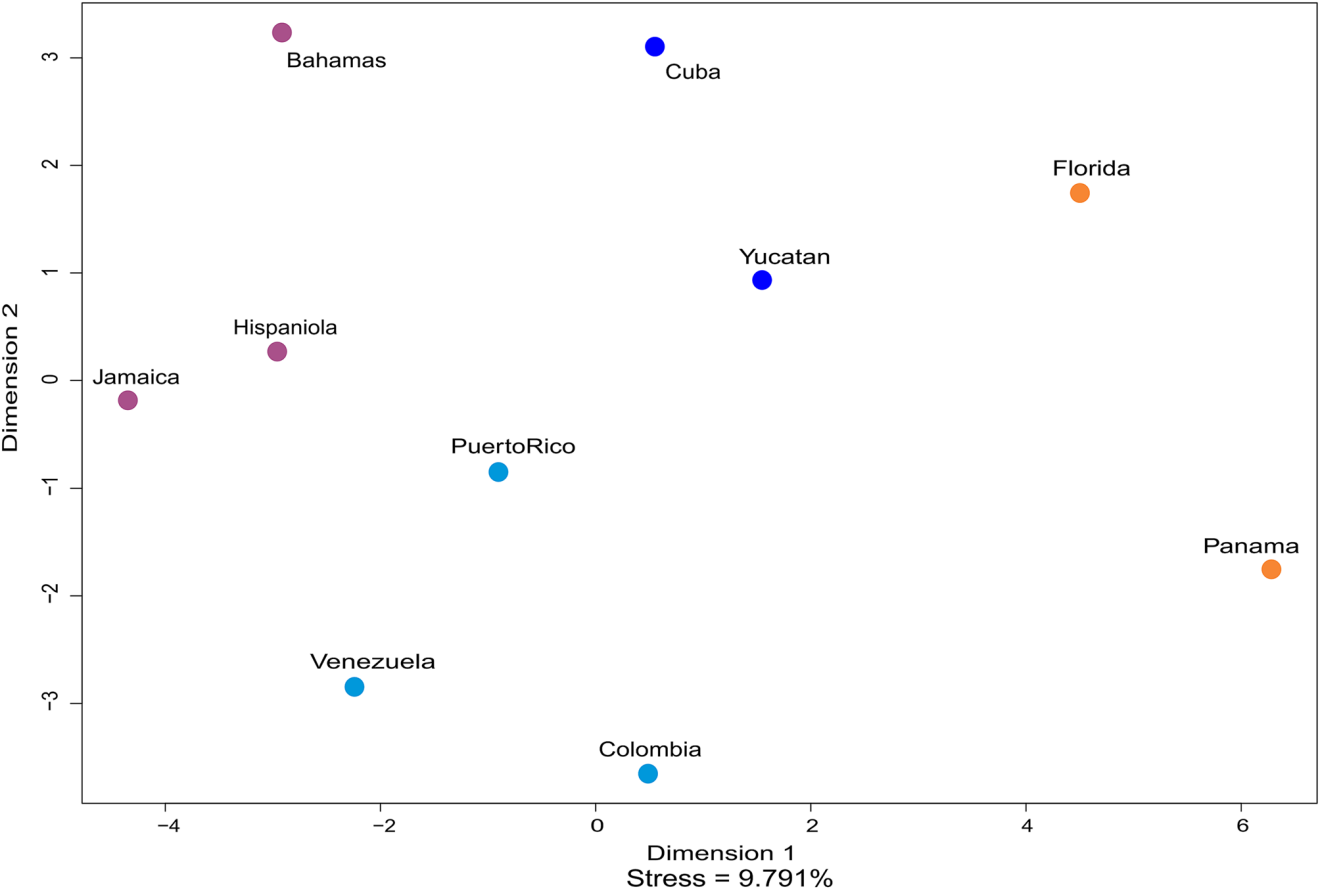
Morphological affinities among the series according to Multidimensional Scaling Analysis of the Mahalanobis Distances reported by Ross et al.^1^ for 103 pre-Columbian individuals from 10 localities. Series colours follow the four clusters identified in Ross et al.^1^, Fig. 5.

The MDS in Fig. 1 shows that Puerto Rico is in fact closer to Hispaniola and Jamaica than to the South American series, which is coherent with the D^2^ values (Ross et al.^1^ Table 3), thus indicating the smallest biological distances to the Puerto Rico series are indeed Jamaica and Hispaniola. Likewise, Florida exhibits similar distances to Yucatán, Cuba, and Panama in the MDS. While the smallest biological distance to Florida in Table 3 of Ross et al.^1^ is with Panama (6.10), the similar distances to Yucatán (6.66) and Cuba (6.58) cannot support the existence of a basal cluster between Florida and Panama, as suggested by their cluster analysis. The MDS thus represents the morphological distances among series with significantly less bias, controverting Ross et al.’s^1^ conclusions that “individuals from Florida and Panama…show no clear relationship with individuals from the islands” (p. 4), and that “data confirm a biological relationship between individuals from Venezuela and Puerto Rico” (p. 4). Further, these conclusions are undermined by not examining the clustering dendrogram for treeness; not providing confidence measures for the purported nodes; and not acknowledging that canonical variate analysis artificially creates groups, especially with samples as small as these^8,9^.

Additionally, in the absence of a demonstrated biological connection to western Venezuela the HJB cluster’s link to this region hinges solely on the claim that “Meillacoid pottery [from Hispaniola, Jamaica, and the Bahamas] is identical to the pottery associated with the Carib expansion” (p. 4)^1^ in the Amazon and Orinoco Basins. No archaeological evidence, such as shared pottery motifs or modes, is provided to support this statement save a reference to Lathrap^10^, who does not actually discuss Meillacoid pottery in his volume. Because Ross et al.^1^ do not specify archaeological correlates, their proposed Carib-HJB migration cannot be evaluated independently based on the artefactual record. The idea that Meillacoid pottery is Carib in origin appears to stem primarily from arguments published by Zucchi^11^ 30 years prior, who suggested that it arose from a multi-ethnic merger of continental Arawak (Cedeñoid tradition) and Carib (Arauquinoid-Valloid traditions) groups based on overly general stylistic similarities (see also Veloz Maggiolo et al.^12^).

To further corroborate a western Venezuelan-HJB connection, Ross et al.^1^ point to the Antillean presence of guinea pigs (*Cavia porcellus*) with a Colombian genetic signature^13^. This misrepresents the archaeological record. Of the 64 guinea pig individuals identified across the pre-contact Caribbean, 81% occur in Puerto Rico and the Virgin Islands—where Meillacoid pottery does not occur—in sites associated with Arawakan-speakers^14^. Only five individuals are known from Hispaniola and Jamaica and none from the Bahamas^14^. This pattern is inconsistent with the Carib migration hypothesis as it indicates initial guinea pig introduction(s) east of Hispaniola and subsequent outward spread, in line with more recent next generation sequencing of ancient Caribbean guinea pig mitogenomes^15^.

Finally, we harbour concerns regarding Ross et al.’s^1^ inappropriate association of human phenotypic variation based on cranial morphology with Island Carib ethnic identities and behavioral tropes constructed by early European chroniclers. Apart from sharing the same origin in the expansion of South American Cariban language groups, the proposed Carib-HJB migrants are archaeologically unrelated to the Island Caribs, who occupied the southern Lesser Antilles at European contact and were the subject of Columbus’ *Diario* accounts of war-mongering cannibals^16^. Scholars have questioned these European narratives of cannibalism, suggesting instead that they were convenient structuring devices employed by the Spanish in furthering their political and economic ambitions and are not supported by archaeological evidence^17–22^. It is unclear why Ross et al. draw on these sensational narratives, especially since their study excludes the Lesser Antilles. Their first paragraph emphasizes the Spanish conceptualization of the Caribs as intrinsically violent, reinforced by references to “invasion”/ “invaders” (pp. 1, 3, 4 in Ross et al.^1^) and Theodore de Bry’s illustration of indigenous *Brazilians* consuming human flesh (Fig. 1 in Ross et al.^1^). This language reproduces bias and reanimates colonial metonymies, providing tacit endorsement for the long-discredited idea that ethnic groups and behavioral traits overlap. We find this especially concerning as it may harm contemporary Island Carib descendant communities (the Kalinago of the Lesser Antilles and Garifuna [also known as the Black Caribs] of Central America) and South American communities, who, through either their connection with the Cariban language family or “Carib” designation, are affected by claims of Carib cannibalism. More broadly, it is highly questionable whether the identified craniometric clusters capture meaningful social differentiation amongst Caribbean Amerindians. While Hispaniolan, Jamaican, and Bahamian peoples may have been similar in facial appearance, it does not follow that they self-identified as a cohesive (Carib) ethnic group. Identities based on highly contingent ethnic frameworks often map poorly onto patterning in phenotypic heterogeneity^23^.

In sum, Ross et al.’s^1^ study is limited by sampling issues; problematic methodological choices; poorly supported archaeological claims; and inappropriate conflations of biology, ethnicity, artefacts, and questionable historical accounts. The authors have extended their interpretations beyond the limits of the available GMM and archaeological data. In our view, there is no evidence to conclude that Hispaniola, Jamaica, and the Bahamas were “invaded” by Carib migrants from western Venezuela. Our critique is consistent with the results of two genome-wide studies of 93^24^ and 184^25^ precontact Caribbean and circum-Caribbean individuals published after Ross et al.’s^1^ study, which also provided no support for a Carib migration to the Greater Antilles.

## Data Availability

All data analysed for this reply were obtained from the Ross et al.^1^ original article.
